# Transcriptomic profiling reveals disease-specific characteristics of epithelial cells in idiopathic pulmonary fibrosis

**DOI:** 10.1186/s12931-020-01414-z

**Published:** 2020-06-30

**Authors:** Maximilian Boesch, Florent Baty, Martin H. Brutsche, Michael Tamm, Julien Roux, Lars Knudsen, Amiq Gazdhar, Thomas Geiser, Petra Khan, Katrin E. Hostettler

**Affiliations:** 1 Lung Center, Cantonal Hospital St. Gallen, Rorschacherstrasse 95, CH-9007 St.Gallen, Switzerland; 2 Department of Biomedicine, University Hospital Basel, University of Basel, Hebelstrasse 20, CH-4031 Basel, Switzerland; 3 Clinics of Respiratory Medicine, University Hospital Basel, University of Basel, Basel, Switzerland; 4 Swiss Institute of Bioinformatics, Basel, Switzerland; 5 Institute of Functional and Applied Anatomy, Hannover Medical School, Hannover, Germany; 6 Department of Pulmonary Medicine, University Hospital Bern, Bern, Switzerland

**Keywords:** Idiopathic pulmonary fibrosis, Lung fibrosis, Epithelial cell, Mesenchymal stem cell, RNA-sequencing

## Abstract

**Background:**

Idiopathic pulmonary fibrosis (IPF) is an incurable disease characterized by progressive lung fibrosis ultimately resulting in respiratory failure and death. Recurrent micro-injuries to the alveolar epithelium and aberrant alveolar wound healing with impaired re-epithelialization define the initial steps of the pathogenic trajectory. Failure of timely alveolar epithelial repair triggers hyper-proliferation of mesenchymal cells accompanied by increased deposition of extracellular matrix into the lung interstitium.

**Methods:**

We previously isolated fibrosis-specific mesenchymal stem cell (MSC)-like cells from lung tissue of patients with interstitial lung diseases. These cells produced factors bearing anti-fibrotic potential and changed their morphology from mesenchymal to epithelial upon culture in an epithelial cell (EC)-specific growth medium. Here, we set out to molecularly characterize these MSC-like cell-derived ECs using global gene expression profiling by RNA-sequencing. Moreover, we aimed at characterizing disease-specific differences by comparing the transcriptomes of ECs from IPF and non-IPF sources.

**Results:**

Our results suggest that differentially expressed genes are enriched for factors related to fibrosis, hypoxia, bacterial colonization and metabolism, thus reflecting many of the hallmark characteristics of pulmonary fibrosis. IPF-ECs showed enrichment of both pro- and anti-fibrotic genes, consistent with the notion of adaptive, compensatory regulation.

**Conclusions:**

Our findings support the hypothesis of a functional impairment of IPF-ECs, which could possibly explain the poor clinical outcome of IPF that roughly compares to those of advanced-stage cancers. Our study provides a valuable resource for downstream mechanistic investigation and the quest for novel therapeutic IPF targets.

## Background

Idiopathic pulmonary fibrosis (IPF) is an incurable interstitial lung disease (ILD) characterized by progressive fibrosis and worsening dyspnea, ultimately leading to respiratory failure and death [1, 2]. The prognosis of IPF is markedly poor, with survival rates comparing to those of advanced-stage cancers [2, 3]. Although the underlying pathomechanisms are incompletely understood, it is generally believed that ongoing damage to the alveolar epithelium triggers a secondary fibrotic response in lung-resident fibroblasts that further impinges on lung function [4, 5]; formal support for this concept for IPF pathogenesis came from mechanistic studies in mice [6, 7]. The appreciation of IPF as a predominantly fibrotic, rather than inflammatory, condition [2, 8] has paved the way for new anti-fibrotic medicines including nintedanib [9] and pirfenidone [10].

Recently, we succeeded in isolating MSC-like cells from peripheral human fibrotic lung tissue. The MSC-like cells met the defining criteria of mesenchymal stem cells (MSCs) [11]. Specifically, the cells stained positive for the surface markers CD44, CD90 and CD105, and were able to differentiate into various cell types of the mesenchymal lineage, including adipocytes, osteocytes and chondrocytes. Furthermore, the cells expressed the pluripotency-associated markers Oct-3/4 and Nanog, thus indicating their potential stemness. Of note, MSC-like cells changed their mesenchymal look to a *bona fide *(cobblestone-like) epithelial morphology when cultured in epithelial cell (EC)-specific growth medium. This distinct change in morphology was paralleled by induction of E-cadherin (CD324) expression, a canonical marker of ECs [5].

Here, we sought to determine the transcriptomic differences between these MSC-like cell-derived ECs from IPF and non-IPF sources. We further aimed at delineating the phenotypic properties of these ECs, following the goal to elucidate a possible functional involvement of these cells in IPF, which may help to explain the poor clinical outcome of IPF as compared to other types of ILDs.

## Methods

### Isolation of primary lung-resident MSC-like cells

Characteristics of the study population are specified in Table 1. MSC-like cells were isolated from lung bronchoscopic or surgical biopsies of IPF (*n* = 5) and non-IPF ‘control’ patients (*n* = 5) between November 2015 and November 2016 as previously described [5]. Briefly, the harvested lung tissue was chopped into pieces (~ 1 mm^3^) which were then cultured in tissue flasks under standard conditions (37 °C, 21% O_2_, 5% CO_2_) in DMEM supplemented with fetal bovine serum (10%), penicillin (20 U/l), streptomycin (20 μg/ml), and amphotericin B (2.5 μg/ml). MSC-like cells showed sprouting-like growth emanating from the tissue samples and finally formed a confluent cell monolayer around the tissue piece. The non-adherent cell fraction was washed away over the course of the culture through repeated exchange of medium. After 5 days, the tissue pieces were removed and the medium was exchanged for the EC-specific growth medium Cnt-17 (CELLnTEC Advanced Cell Systems AG, Bern, Switzerland). Total RNA was extracted after 7 days of culture in Cnt-17.

**Table 1:** Patient Characteristics (*n* = 10)

Patient ID	Sex	Age (years)	Clinical Diagnosis
**IPF (*n*** **= 5)**			
001	Male	65	IPF
002	Male	63	IPF
003	Female	49	IPF
004	Male	77	IPF
005	Male	60	IPF
**Non-IPF (*n*** **= 5)**			
006	Male	37	Non-classifiable ILD
007	Male	67	Vasculitis-associated ILD
008	Male	71	Non-classifiable ILD
009	Female	75	Carcinoma
010	Male	72	Chronic eosinophilic pneumonia

*Abbreviations used: IPF* Idiopathic pulmonary fibrosis; *ILD* Interstitial lung disease

### RNA extraction

Total RNA was extracted from ECs (bulk populations) using the MicroElute Total RNA Kit (Omega Bio-Tek, Norcross, GA). RNA was quantified using the QuantiFluor RNA System (Promega, Madison, WI) and quality-controlled on the Bioanalyzer instrument using the RNA 6000 Pico Kit (both from Agilent, Santa Clara, CA).

### RNA-sequencing

Fifty ng total RNA were used for library generation using the TruSeq Stranded mRNA Library Prep Kit High Throughput (Illumina, San Diego, CA), and 15 PCR cycles were performed. The Standard Sensitivity NGS Fragment Analysis Kit (Advanced Analytical, Ames, IA) was used to assess the quality of the libraries (123 ± 30 nmol/L average concentration and 321 ± 8 bp average library size). 1.8 pM of pooled sample was used for cluster generation on the NextSeq 500 instrument (Illumina). Single-read 76 bases (plus 8 + 8 bases for indexing) were sequenced using the NextSeq 500 High Output Kit 75-cycles (Illumina). Primary analysis of sequencing data was conducted using RTA version 2.4.11 and bcl2fastq v2.20.0.422 (both from Illumina).

### qPCR validation

Total RNA was isolated from additional samples of an independent cohort and gene expression levels were determined using TaqMan® Gene Expression Assays (Thermo Fisher Scientific, Waltham, MA). The following target genes were analyzed: CXCL8 (Hs00174103 m1), ICAM1 (Hs00164932 m1), PTGS2 (Hs00153133 m1), LIF (Hs01055668 m1), IL7R (Hs00902334 m1), and GAPDH/housekeeper (Hs03929097_g1). Reactions were performed at 50 °C for 2 min (1 cycle), 95 °C for 10 min (1 cycle), and 95 °C for 15 s followed by 60 °C for 1 min (40 cycles). Relative expression data are shown as -ΔCt of target gene-GAPDH.

### Statistical and bioinformatic considerations

All analyses were done using the R statistical software (R Core Team 2018, www.R-project.org). The Bioconductor package ‘edgeR’ (version 3.24.3) was used for the identification of differentially expressed genes. Exploratory analyses including Principal Component Analysis (PCA) were performed using the package ‘ade4’. Pathway enrichment analyses and visualization were done using the package ‘clusterProfiler’ (version 3.10.1) [12]. Differentially expressed gene overrepresentation in various functional annotation databases including Gene Ontology (GO, http://geneontology.org), Kyoto Encyclopedia of Genes and Genomes (KEGG, www.genome.jp/kegg/pathway.html) and DisGeNET (www.disgenet.org/rdf) was investigated using hypergeometric tests as implemented in clusterProfiler.

## Results

### Transcriptomic profiling of ECs

**Fig. 1:**
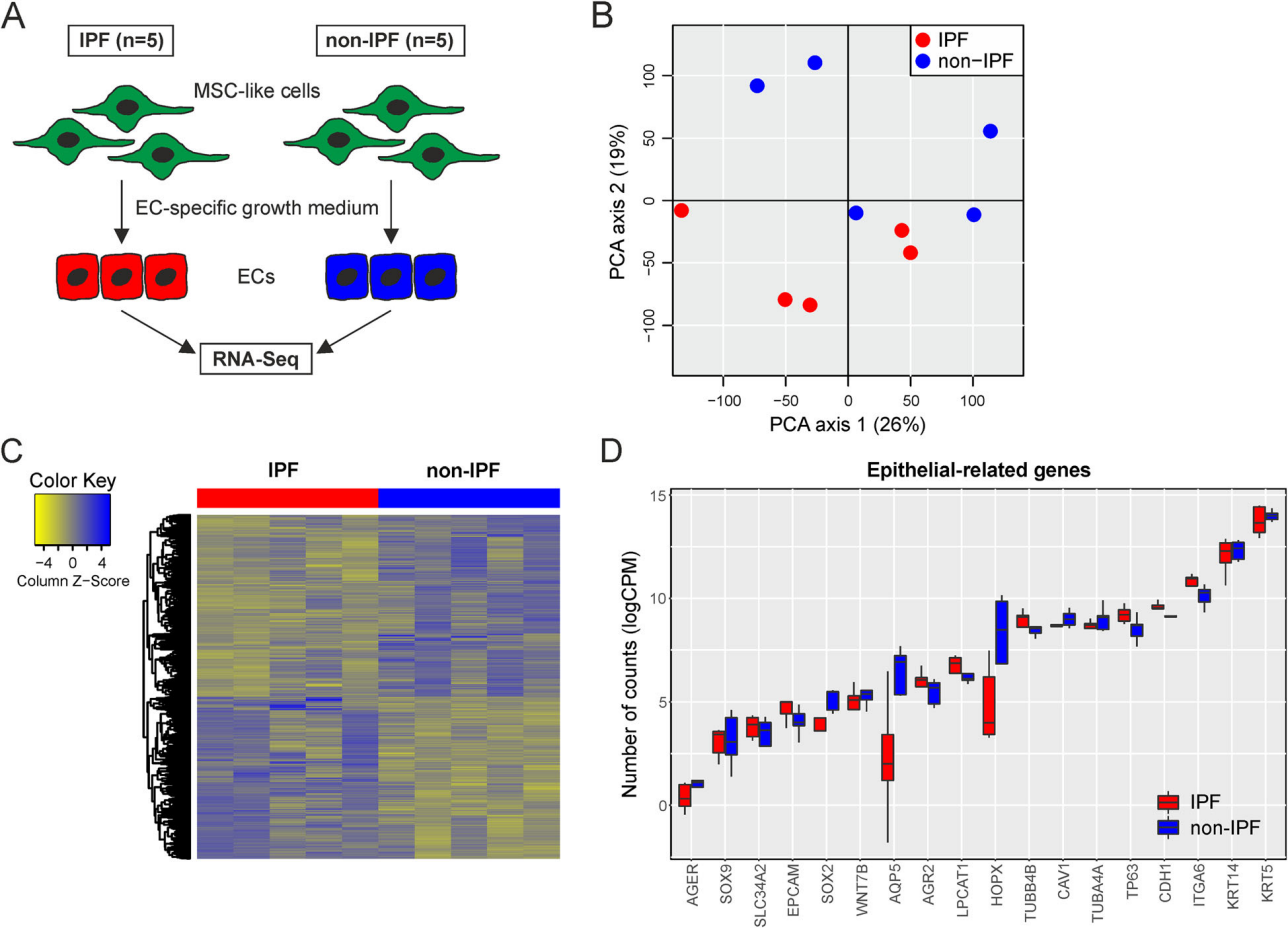
RNA-Sequencing of MSC-Like Cell-Derived ECs. **a** Conceptual and experimental design of the study. **b** PCA scores of ECs from IPF and non-IPF sources. **c** Heatmap representation and hierarchical cluster analysis of global gene expression in IPF *versus *non-IPF samples. A total of 199 genes were differentially regulated in IPF versus non-IPF samples. **d** Expression of epithelial-related markers in ECs from IPF and non-IPF sources. *Abbreviations used:* CPM, counts per million (reads mapped); EC, epithelial cell; IPF, idiopathic pulmonary fibrosis; MSC, mesenchymal stem cell; PCA, Principal Component Analysis

We isolated primary MSC-like cells from bronchoscopic or surgical biopsies of patients with IPF (*n* = 5) and non-IPF lung disease (‘control’; *n* = 5) (Table 1). While we previously showed that MSC-like cells exhibit EC morphology when cultured in an EC-specific growth medium [5], we here set out to molecularly characterize these ECs using global expression profiling by RNA-sequencing (Fig. 1a). Despite quite some variation between samples, PCA separated IPF from non-IPF samples on the two first principal components (Fig. 1b), indicating disease-specific transcriptomic differences. A differential expression analysis revealed the differential expression of 199 genes between IPF and non-IPF (FDR-adjusted *p*-values < 0.05) (Fig. 1c). Of note, epithelial markers including EPCAM, CDH1 (encoding E-cadherin/CD324) and keratins displayed only minor, non-statistically significant differences between IPF and non-IPF (FDR-adjusted *p*-values > 0.05) (Fig. 1d).

### Principal signatures of ECs from an IPF source

**Fig. 2:**
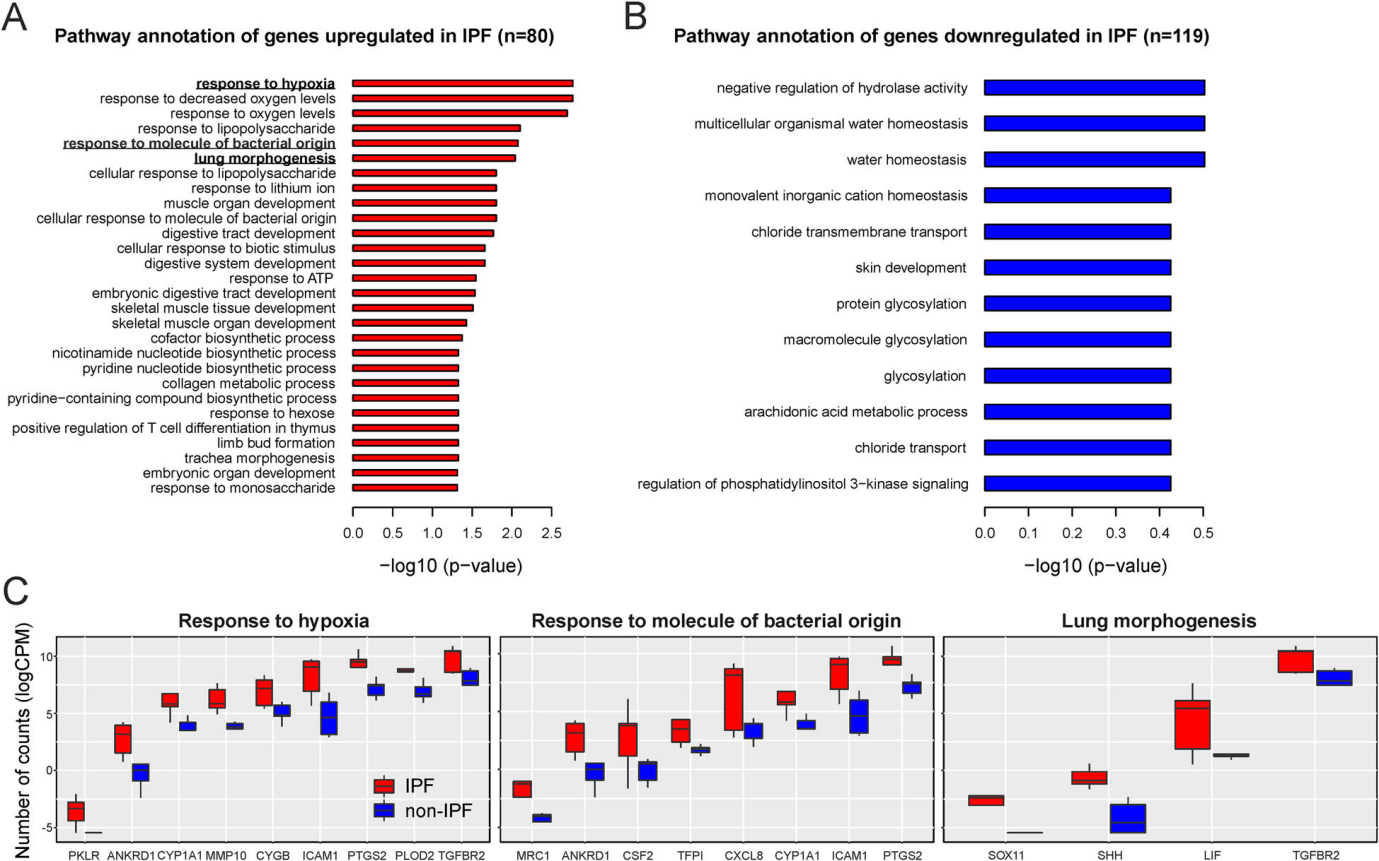
Functional Pathway Annotation Using GO Enrichment Analysis. Functional enrichment analysis of upregulated (**a**) and downregulated pathways (**b**) in IPF-ECs using GO annotations. (**c**) In-depth analysis of three upregulated pathways that likely play a role in IPF pathogenesis. *Abbreviations used:* CPM, counts per million (reads mapped); EC, epithelial cell; GO, Gene Ontology; IPF, idiopathic pulmonary fibrosis

**Fig. 3:**
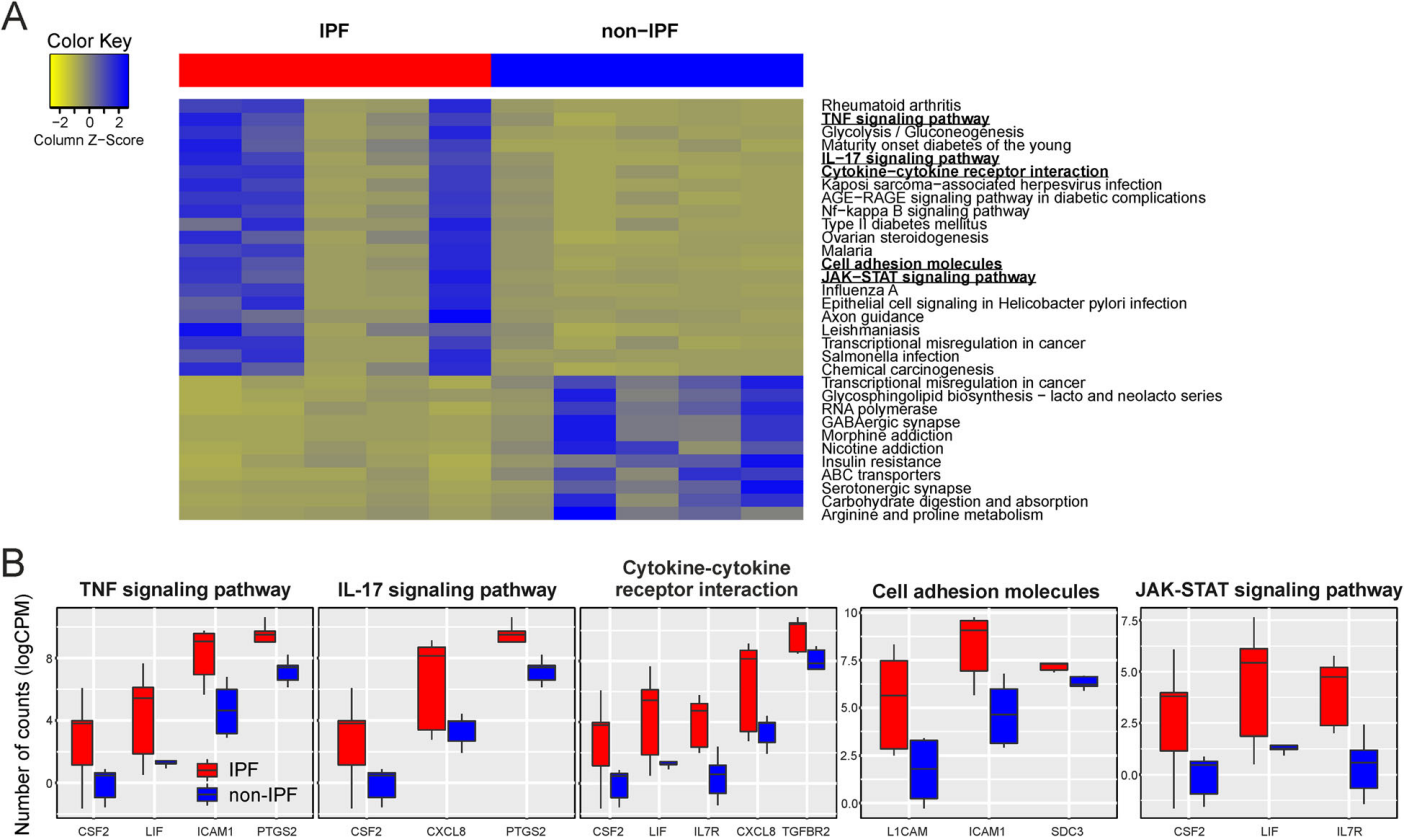
Functional Pathway Annotation Using KEGG Enrichment Analysis. **a** Heatmap analysis of functional pathway enrichment in IPF *versus* non-IPF samples using KEGG annotations. **b** In-depth analysis of five immune-related pathways that demonstrate upregulation in ECs from an IPF source. *Abbreviations used:* CPM, counts per million (reads mapped); EC, epithelial cell; IPF, idiopathic pulmonary fibrosis; KEGG, Kyoto Encyclopedia of Genes and Genomes

**Fig. 4:**
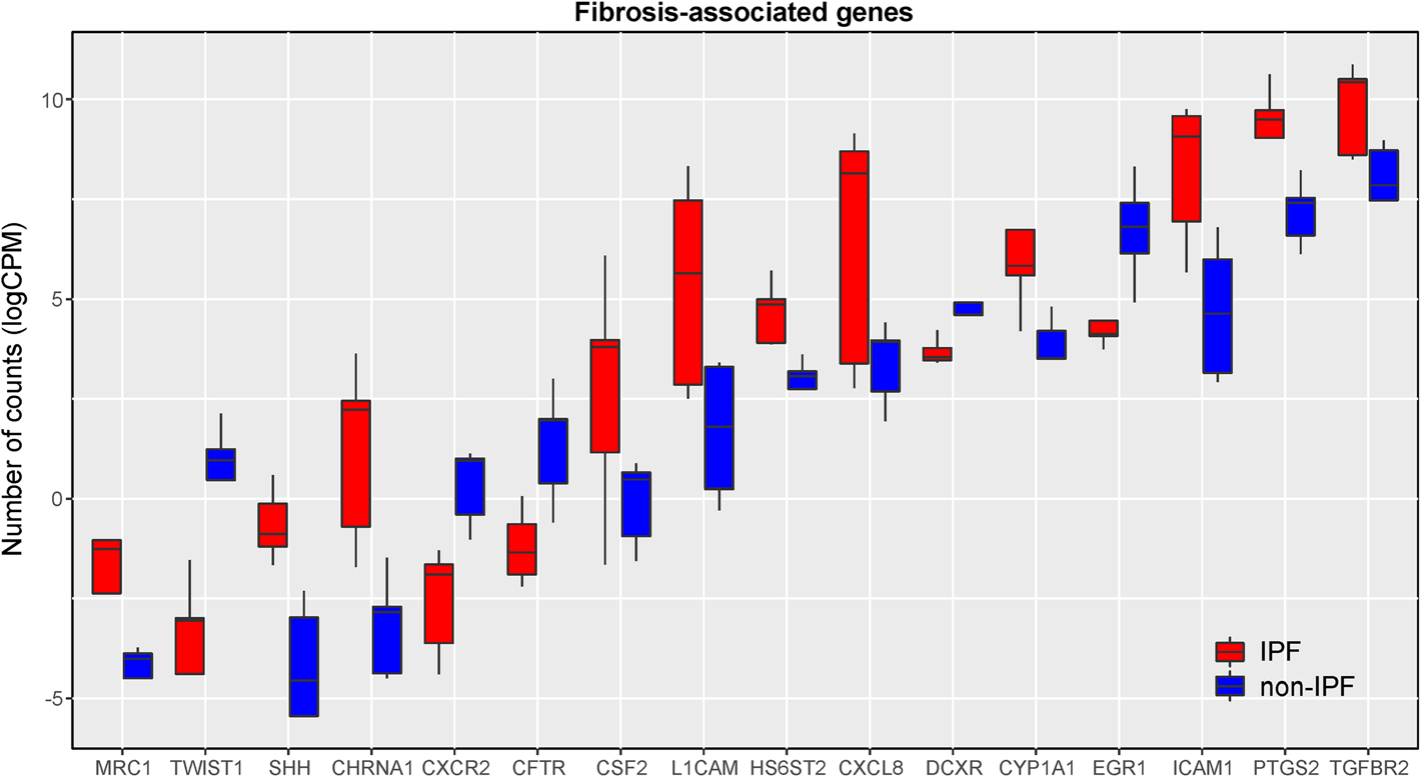
Expression Pattern of Fibrosis-Associated Genes in ECs. Comparative bar plot representation of the expression of 16 fibrosis-associated genes in IPF *versus* non-IPF samples. The gene panel was retrieved from the DisGeNET database. *Abbreviations used:* CPM, counts per million (reads mapped); EC, epithelial cell; IPF, idiopathic pulmonary fibrosis

We annotated the up- (*n* = 80) and downregulated genes in IPF (*n* = 119) (**Supplementary Table** **1**) using functional enrichment analysis on the biological processes of the GO database. We found that IPF-ECs were enriched in various pathways potentially related to IPF pathogenesis including signatures specific for hypoxia, response to microbes, and organ development (Fig. 2a). Conversely, pathways related to ion homeostasis/transport as well as glycosylation were enriched for downregulated genes (Fig. 2b). We specifically analyzed the differentially expressed genes with respect to gene sets/pathways that likely play a role in the development and/or progression of IPF – ‘response to hypoxia’, ‘response to molecule of bacterial origin’ and ‘lung morphogenesis’ (Fig. 2c). Between 4 and 9 differentially expressed genes could be allocated to these pathways (of which some were overlapping between the signatures) and included adhesion molecules (ICAM1), chemokines (CXCL8), growth factors (CSF2), growth factor receptors (TGFBR2), matrix metalloproteinases (MMP10), and stem cell-related factors (SOX11, SHH, LIF). Notably, some of the genes were previously reported to be involved in pulmonary fibrosis pathogenesis [13–15]. We next performed functional enrichment analysis of the differentially expressed genes using the KEGG pathway database. Although inflammation is no longer considered the main driving force behind IPF development and progression [2, 8], this analysis showed enrichment of various immune-related pathways in IPF-ECs, including the TNF, IL-17, and JAK-STAT signaling pathways (*p* < 0.05) (Fig. 3a+b). It should be noted, though, that these signatures largely involved the same differentially expressed genes as the enriched GO categories (Fig. 2). Moreover, quite some variation was observed within the IPF and non-IPF groups (Fig. 3a), consistent with the notion of significant interpatient variability. Using the DisGeNET database to analyze the differentially expressed genes with regards to IPF hallmark characteristics, we found evidence that ECs from an IPF source (‘IPF-ECs’) expressed fibrosis-associated genes, suggestive of persistent ‘imprinting’ from the original IPF milieu ex vivo (Fig. 4). We next set out to validate key genes from the RNA-sequencing experiment using qPCR analysis of independent IPF and control patients (**Supplementary Table** **2**). As shown in **Supplementary Figure** **1****A**, four out of five key genes (CXCL8, ICAM1, LIF, PTGS2) could be validated as overexpressed in IPF, with the remaining gene (IL7R) showing enrichment in IPF samples as well without reaching statistical significance. Moreover, comparing these key genes to the publically available GSE134692 dataset [16], we found that three of the genes (CXCL8, LIF, PTGS2) showed the same direction of regulation among IPF and non-IPF samples, whereas ICAM1 and IL7R were differentially regulated between the datasets (**Supplementary Figure** **1****B**). This suggested both similarities and characteristic differences between the datasets, thus corroborating our findings but also highlighting the distinct nature of our ex vivo model in comparison to primary explanted tissue. Moreover, the increasing appreciation of senescence as a major contributor to IPF development [17] prompted us to specifically investigate the KEGG pathway ‘cellular senescence’. However, of the 160 genes allocated to this pathway, only two (CXCL8 and TGFBR2) were found to be overlapping with our dataset, suggesting that this cellular process plays only a minor part in IPF-ECs. Taken together, these data suggest that the molecular signature of MSC-like cell-derived ECs may at least partially reflect the biological situation in the IPF-affected lung, including the hallmark characteristics (i) compromised gas exchange, (ii) aberrant bacterial colonization/dysbiosis, and (iii) developmental remodeling/repair.

### Functional pathway analysis reveals IPF-specific signaling circuits

**Fig. 5:**
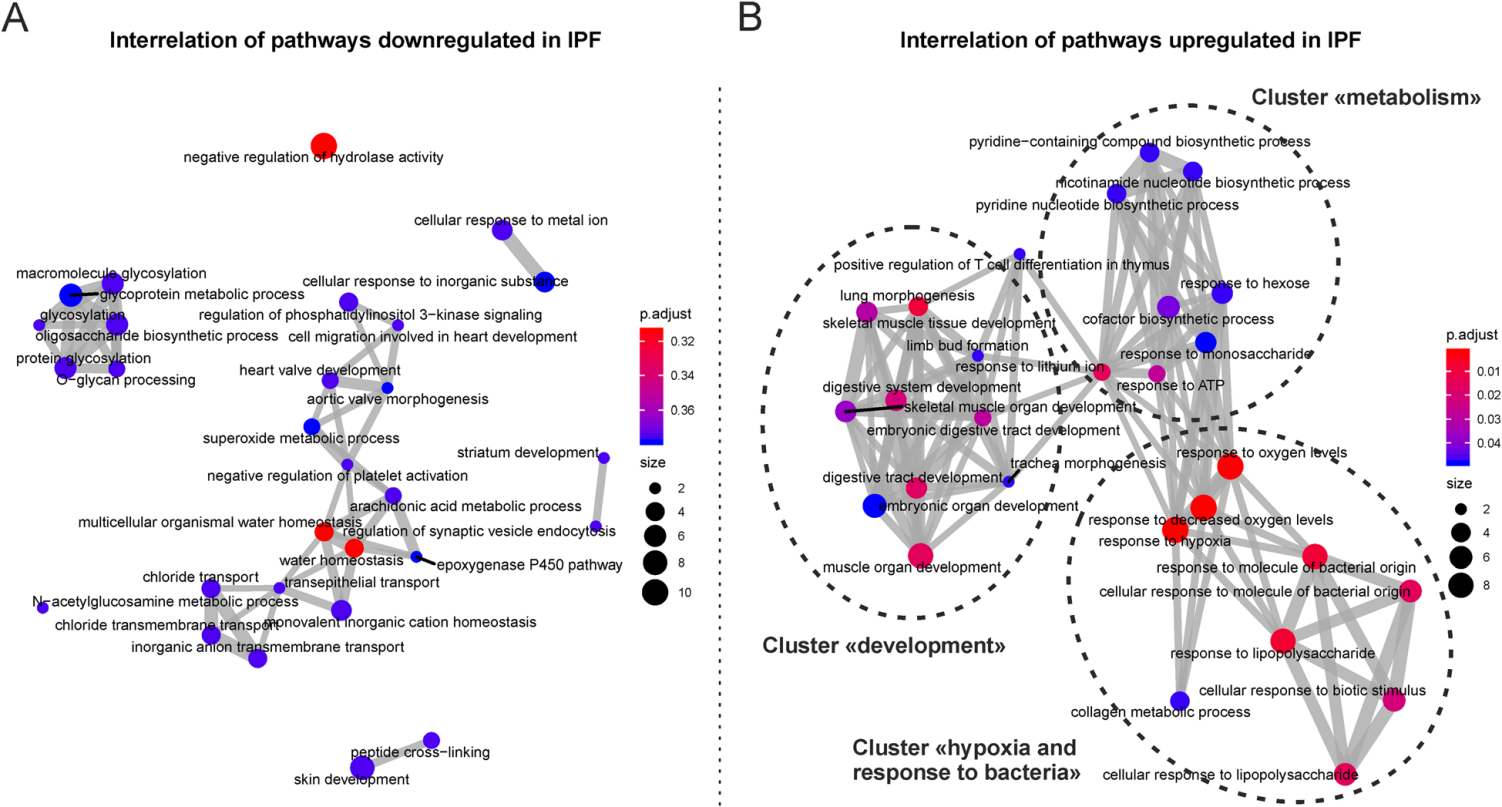
Analysis of Pathway Interrelation. Network analysis of functional pathways downregulated (**a**) and upregulated (**b**) in ECs from an IPF source using the R package ‘clusterProfiler’. Significant clustering was manually delineated and the clusters were named based on their integrated biological processes (B). *Abbreviations used:* EC, epithelial cell; IPF, idiopathic pulmonary fibrosis

We moved on to interrogate the interconnection of the differentially regulated pathways and biological processes using the R package ‘clusterProfiler’. While pathways enriched for downregulated genes in IPF-ECs did not notably cluster (Fig. 5a), activated pathways showed functional enrichment mostly within three clusters that we termed ‘development’, ‘hypoxia and response to bacteria’, and ‘metabolism’ (Fig. 5b). Within the cluster ‘development’, several organ-specific developmental pathways were identified, suggesting overlapping mechanisms of tissue remodeling occurring in the IPF lung and during embryonic development. In addition, enrichment for development-related genes and pathways may indicate stem cell potential of the ECs – in turn necessary for epithelium-regenerating capacity. While the cluster ‘metabolism’ may reflect the metabolic requirements of a disease with a proliferative character, the cluster ‘hypoxia and response to bacteria’ may be more specific for IPF and reflect the typical conditions in the IPF-affected lung, including low oxygen levels and dysbiosis. Moreover, the pathway ‘collagen metabolic process’ within the same cluster may indicate the overt fibrotic response in the diseased lung characterized at least in part by massive collagen matrix deposition. Collectively, comprehensive analysis of the interrelation of pathways revealed distinct functional clusters that are plausibly implicated in the pathogenesis of IPF.

## Discussion

In this article, we provide a comprehensive description of the transcriptomic landscape of MSC-like cell-derived ECs from IPF *versus* non-IPF sources. Results from this study suggest that IPF-ECs (i) remain imprinted from the in vivo IPF milieu (hypoxia and dysbiosis), (ii) are more fibrotic than ECs from non-IPF sources, (iii) are enriched in various immune-related pathways, and (iv) carry signatures associated with organ development and cellular stemness. We therefore propose a distinct functional failure of these cells in IPF, which may help to explain the poor clinical outcome of IPF patients.

It is important to note that ECs were experimentally generated using a specific cell culture method and that their in vivo relevance is yet to be determined. Overlapping findings with the GSE134692 dataset based on primary tissue specimens from lung transplant recipients and donors [16] suggest, however, that our model may be able to capture the principal signatures of the IPF-affected lung including upregulation of CXCL8, LIF, and PTGS2. CXLC8 (encoding interleukin 8) is an inflammatory mediator attracting neutrophils and contributing to pathogen clearance, but may also confer secondary fibrotic tissue damage [18, 19]. In contrast, PTGS2 (encoding cyclooxygenase-2) [20, 21] and LIF [22] are believed to counteract fibrosis through various mechanisms including ferroptosis and immune modulation. Their upregulation as seen here may therefore indicate compensatory anti-fibrotic activity of the MSC-like cell-derived ECs that warrants future investigation.

IPF is an age-related disease characterized by progressive and irreversible scarring [23, 24]. The predominant occurrence in the elderly population is in line with the idea that cellular senescence drives, and is ultimately responsible for, pulmonary fibrosis [17]. While senescent fibroblasts have been shown to be fibrogenic and causally involved in disease pathogenesis in the bleomycin-injury IPF model [17], other studies have suggested a role for alveolar EC senescence in triggering lung remodeling and fibrosis [7, 25]. Mechanistically, adoption of a senescent phenotypic state may result from mutations in the enzyme telomerase reverse transcriptase, shortened telomeres, or other telomere dysfunctions [7, 26]. The senescence of functional (ECs) and/or structural (fibroblasts) lung cells is thought to be ‘counteracted’ by increased proliferation of non-senescent fibroblasts, with little success, finally resulting in excessive matrix deposition and fibrosis/scarring [27]. Indeed, we have looked at signatures of cellular senescence without detecting relevant enrichment of senescence-associated genes in IPF-ECs. We speculate that this may be explainable by their derivation from lung-resident stem-like cells rather than alveolar ECs or fibroblasts.

Autophagy, i.e., the on-demand degradation and recycling of intracellular components and organelles, is yet another fundamental cell process that is deregulated in IPF. Diminished also during aging, the autophagy flux is typically reduced to insufficient levels in IPF, which in turn accelerates cellular senescence and provokes pro-fibrotic myofibroblast differentiation [28, 29]. As these effects appear to be mediated through TGFβ signaling, upregulation of TGFBR2 in IPF-ECs may indicate IPF-specific perturbation in the autophagy cascade.

Our study is based on fresh, native samples from a rare patient cohort and hence provides a valuable resource for IPF research. It is important, however, to mention also the limitations of our study: First, our study is based on a limited number of cases, which precludes that robust, general conclusions can be drawn. Second, the reference cohort was based on patients with various kinds of non-IPF lung diseases, rather than healthy individuals. Third, our study is descriptive, meaning that causal links or rational therapeutic targets cannot be established. Fourth, our study is ultimately based on a single technological platform, i.e., RNA-sequencing, and does not report protein-level data.

## Conclusion

Taken altogether, we here provide a comprehensive, molecular characterization of IPF-ECs generated ex vivo from IPF patients. Our results suggest that the transcriptome of IPF-ECs is clearly distinct from non-IPF-ECs and dominated by signatures whose underlying biological processes are plausibly involved in IPF pathogenesis, such as hypoxia, bacteria, metabolism and development. Further research is warranted to decipher the suitability of ECs as a cell-based medicinal product for anti-fibrotic, epithelium-regenerating treatment of IPF.

## Supplementary information

**Additional file 1: Supplementary Table 1.** List of Differentially Expressed Genes. A total of 199 genes were differentially expressed in MSC-like cell-derived ECs from IPF *versus* non-IPF patients, with 80 genes upregulated and 119 genes downregulated in IPF-ECs. *Abbreviations used:* CPM, counts per million (reads mapped); EC, epithelial cell; FC, fold change; FDR, false discovery rate; IPF, idiopathic pulmonary fibrosis; MSC, mesenchymal stem cell.

**Additional file 2: Supplementary Table 2.** Patient Characteristics of qPCR Validation Cohort (*n* = 16). *Abbreviations used:* IPF, idiopathic pulmonary fibrosis; ILD, interstitial lung disease.

**Additional file 3: Supplementary Figure 1.** qPCR Validation of RNA-Sequencing Results and Comparison to a Published Dataset. (A) TaqMan-based qPCR analysis of 5 key genes in independent cohorts of IPF (*n* = 9) and non-IPF patients (*n* = 7; one patient overlapping with the RNA-sequencing analysis). (B) Expression levels of the 5 key genes in IPF and non-IPF patients from the GSE134692 dataset and two-tiered comparison to our data (same direction of regulation – yes/no). *Abbreviations used:* CPM, counts per million (reads mapped); IPF, idiopathic pulmonary fibrosis.

## Data Availability

Processed RNA-sequencing data have been deposited in NCBI’s Gene Expression Omnibus (GEO) and are accessible through GEO series accession number GSE151673.

## Abbreviations

EC: Epithelial cell
FDR: False discovery rate
GO: Gene ontology
ILD: Interstitial lung disease
IPF: Idiopathic pulmonary fibrosis
KEGG: Kyoto encyclopedia of genes and genomes
MSC: Mesenchymal stem cell
PCA: Principal component analysis
