# Supplementary material for: Transcriptomic profiling reveals disease-specific characteristics of epithelial cells in idiopathic pulmonary fibrosis
Source: Respir Res. 2020 Jun 30;21:165. doi: 10.1186/s12931-020-01414-z (PMC7329456; doi:10.1186/s12931-020-01414-z)
Supplement: Supplementary file 2 — Additional file 2: Supplementary Table 2. Patient Characteristics of qPCR Validation Cohort (n = 16). Abbreviations used: IPF, idiopathic pulmonary fibrosis; ILD, interstitial lung disease. [file 12931_2020_1414_MOESM2_ESM.docx]

**Supplementary Table 2. Patient Characteristics of qPCR Validation Cohort (n=16).**

| **IPF (n=9)** | | | |
| --- | --- | --- | --- |
| **Patient ID** | **Sex** | **Age (years)** | **Clinical Diagnosis** |
| 011 | Male | 64 | IPF |
| 012 | Male | 59 | IPF |
| 013 | Female | 64 | IPF |
| 014 | Male | 47 | IPF |
| 015 | Male | 60 | IPF |
| 016 | Female | 62 | IPF |
| 017 | Male | 69 | IPF |
| 018 | Female | 79 | IPF |
| 019 | Male | 75 | IPF |
| **Non-IPF (n=7)** | | | |
| **Patient ID** | **Sex** | **Age (years)** | **Clinical Diagnosis** |
| 008 | Male | 71 | Non-classifiable ILD |
| 020 | Female | 88 | Hypersensitivity pneumonitis |
| 021 | Female | 79 | Sjörgen-associated ILD |
| 022 | Male | 70 | Organizing pneumonia |
| 023 | Male | 75 | MPO-ANCA-associated ILD |
| 024 | Male | 73 | Rheumatoid arthritis-associated ILD |
| 025 | Female | 79 | Chronic hypersensitivity pneumonitis |

*Abbreviations used:* IPF, idiopathic pulmonary fibrosis; ILD, interstitial lung disease.
