# Supplementary figures and images for: Transcriptomic profiling reveals disease-specific characteristics of epithelial cells in idiopathic pulmonary fibrosis
Source: Respir Res. 2020 Jun 30;21:165. doi: 10.1186/s12931-020-01414-z (PMC7329456; doi:10.1186/s12931-020-01414-z)

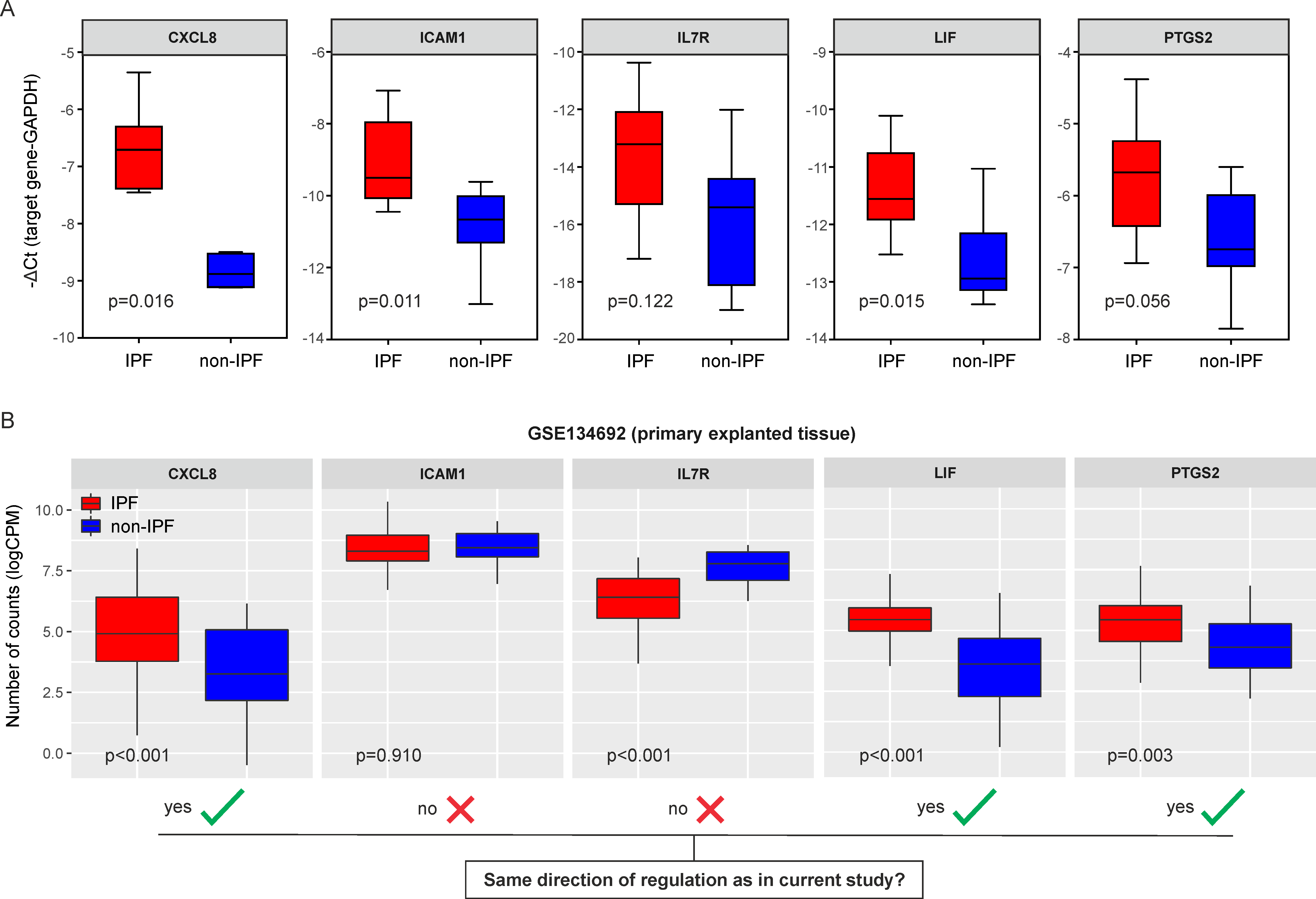

Supplement: Supplementary file 3 — Additional file 3: Supplementary Figure 1. qPCR Validation of RNA-Sequencing Results and Comparison to a Published Dataset. (A) TaqMan-based qPCR analysis of 5 key genes in independent cohorts of IPF (n = 9) and non-IPF patients (n = 7; one patient overlapping with the RNA-sequencing analysis). (B) Expression levels of the 5 key genes in IPF and non-IPF patients from the GSE134692 dataset and two-tiered comparison to our data (same direction of regulation – yes/no). Abbreviations used: CPM, counts per million (reads mapped); IPF, idiopathic pulmonary fibrosis. [file 12931_2020_1414_MOESM3_ESM.tif]
